# The strategic targeting of females by transnational tobacco companies in South Korea following trade liberalisation

**DOI:** 10.1186/1744-8603-5-2

**Published:** 2009-01-30

**Authors:** Kelley Lee, Carrie Carpenter, Chaitanya Challa, Sungkyu Lee, Gregory N Connolly, Howard K Koh

**Affiliations:** 1 Centre on Global Change and Health, London School of Hygiene and Tropical Medicine, Keppel Street, London, UK; 2 Division of Public Health Practice, Harvard School of Public Health, Boston, USA; 3 Sophie Davis School of Biomedical Education, City College of New York, New York, USA

## Abstract

**Background:**

In 1988 South Korea opened its cigarette market to foreign companies under the threat of US trade sanctions. Despite strong social stigma against female smoking in South Korea, and restrictions on tobacco marketing to women and children, smoking rates among young Korean females increased from 1.6% in 1988 to 13% in 1998. Previous analyses describe how Asian countries have been targeted by transnational tobacco companies for new markets, with Asian females offering substantial future growth potential. An understanding of the strategies used by TTCs to increase smoking among Korean females is critical to public health efforts to adopt a stronger gender perspective in implementing the Framework Convention on Tobacco Control.

**Methods:**

Internal documents of transnational tobacco corporations were systematically searched using keywords focused on the targeting of the female market since market liberalisation in 1988. Industry documents were analysed alongside primary and secondary data on the tobacco industry in South Korea.

**Results:**

TTCs have targeted Korean females since the late 1980s, conducting market research to understand consumer preferences, cultural characteristics and social changes affecting women and girls. Brands designed to appeal to females have focused on "slim" and "superslim" cigarettes, "light" and "mild" claims, and marketing which appeals to the growing numbers of young women entering the labour force. Strategies for overcoming legal restrictions on marketing to women and children have included the use of company rather than brand names, retail distribution at venues frequented by females, trademark diversification and sponsorship.

**Conclusion:**

Given the high male smoking rates in South Korea, tobacco control efforts have given limited attention to girls and women. The limited data available on female smoking behaviour suggests that, despite legal restrictions and social stigma, smoking among females has increased since market opening, notably within younger age groups. In addition to more detailed trend data, there is an urgent need for the development and implementation of gender-sensitive tobacco control measures. Part of South Korea's accession to the FCTC should include emphasis on measures to address the strategic targeting of Korean females by TTCs.

## Background

The Kobe Declaration, agreed upon at the WHO International Conference on Tobacco and Health in 1999, draws particular attention to the vulnerability of women and girls to the tobacco epidemic. WHO predicts that female smokers will triple in number (from 200 to 600 million by 2025), and the Declaration concludes " [i]t is urgent that we find comprehensive solutions to the danger of tobacco use and address the epidemic among women and girls" [1].

A critical part of the need "to bring gender into the mainstream of tobacco control policies" [2] is fuller understanding of how transnational tobacco companies (TTCs) have strategically targeted women and girls. Industry tactics, during the twentieth century, to increase female smoking are well documented in the US [3] and other "mature markets", are being actively extended to "emerging markets" [4-7]. As the Declaration states, tobacco companies have "launched aggressive campaigns to recruit women and girls worldwide" [8]. In Asia, where a "young women's smoking crisis" [9] is looming, a survey by Bansal et al. reports that cigarette companies in India have developed sophisticated campaigns to target women and children, among others, in different socio-economic groups [10]. In Malaysia and the Philippines, results of a semi-structured questionnaire by Morrow and Barraclough finds that gender is highly significant in cigarette promotion but largely unrecognised in tobacco control policy [11]. Focus group discussions with high school girls in China suggest that "concepts of femininity, independence, style and sophistication are recognised by young women in China as part of the already embedded smoking culture" [12].

This paper reviews internal tobacco industry documents to analyse the strategies used by TTCs to increase tobacco consumption among females in South Korea from the late 1980s. There has been no analysis so far of industry documents in relation to South Korea, one of four Asian countries (along with Japan, Taiwan and Thailand) to open its domestic market to foreign tobacco companies at that time. South Korea has the largest adult male smoking population of all Organisation for Economic Cooperation and Development (OECD) member countries, estimated at 12 million out of 47 million in 2004 [13,14]. Also, as the world's tenth largest economy, the Korean market is described as "one of the most lucrative in the world," with each share point in the highest-priced premium cigarette market generating brand profitability of at least US$25 million [15]. For these reasons, South Korea has been targeted by TTCs as an emerging market requiring "higher priority" [16]. Under Section 301 of the US Trade Act (1974) [17], the US Cigarette Export Association petitioned the US Trade Representative (USTR) to argue that South Korea should remove what were deemed unfair trade barriers to foreign cigarette brands, including high import tariffs and restrictions on marketing and distribution. On 27 May 1988, under the threat of trade sanctions, South Korea signed an agreement with the USTR providing "open, non-discriminatory access to the Korean cigarette market" [18]. Since market opening, transnational tobacco companies (TTCs) have steadily increased their market share in South Korea, from 0.1% (1988) to around 30% (2007) [19]. British American Tobacco (BAT) leads at 16%, followed by Philip Morris International (PMI) at 8.3% and Japan Tobacco International (JTI) at 3% [20]. Correspondingly, the former state-owned monopoly KT&G (Korean Tomorrow and Global, formerly known as Korean Tobacco and Ginseng) has seen its market share decline to around 70% (Table 1).

**Table 1:** Percentage share of cigarette market in South Korea

**Company**	**2003**	**2005**	**2006**	**2007**
Korea Tobacco & Ginseng Corp (KT&G)	76.7	73.0	70.8	69.2

British American Tobacco (BAT)	17.8	18.1	17.3	17.0

Philip Morris International (PMI)	3.5	5.5	8.7	9.8

Japan Tobacco Inc (JTI)	2.0	4.0	3.2	4.0

Sources: Compiled from Ministry of Finance and Economy; Lambat I. Paradigm Shift. *Tobacco Reporter*. June 2006: 28–32; and Yoo SJ. BAT Korea aims to surpass 16% market share. *Korea Herald*. 26 May 2006.

Previously, industry documents have been used to analyse tactics to gain access to emerging markets [21-23], target youth [24], and circumvent tobacco control measures in Asian countries [25-27]. To date, however, documents have not been analysed in detail to understand industry targeting of females in specific Asian countries. Like other Asian countries, historically female smoking rates in South Korea have been low compared with males. Accurate data on female smoking rates remain limited, with measures of prevalence rate distorted by substantial underreporting because of continued social stigma towards female smoking [28]. Nonetheless, available data suggest that smoking rates among females (17–19 years) has risen since the late 1980s, from 1.6% (1988) [18,29] to 13% in 1998–99 [30-32]. Both the tobacco industry and public health advocates predict a continued upward trend in female smoking prevalence [9]. Available data estimates that tobacco caused 46 208 premature deaths in 2003 in South Korea including 6120 female deaths [33,34], costing the country around US$4.6 billion in 1998 [35]. Importantly, this trend has occurred despite restrictions on advertising directed at women and children adopted by the government in 1989 after market opening. For example, tobacco companies are permitted to sponsor social, cultural or sporting events, with the exception of events specifically aimed at women and children. Similarly, cigarette brands can be advertised sixty times per year in the print media, but again publications directed at women and children are excluded [36]. This paper finds that the strategies used by TTCs to target women and girls in South Korea have been developed amid fierce competition for a market share of existing smokers, as well as future growth among younger smokers. Given that the main customers of foreign brands are relatively young [37], the paper describes what products were developed to appeal to the female market, what marketing, advertising and promotion activities were used to encourage consumption and, importantly, how these circumvented existing tobacco control measures. The paper concludes with recommendations for strengthening tobacco control in South Korea and other emerging markets in Asia.

## Methods

This paper analyses tobacco industry documents from the British American Tobacco Documents Archive  and Legacy Tobacco Documents Library . The provenance, mechanics and limitations of using tobacco industry documents have been described elsewhere [38-42], as have the particular difficulties of accessing and working with documents at the Guildford Depository [43-45]. Purposive document searches, undertaken from May 2006 to March 2008, followed an iterative process beginning with broad terms such as "Korea", "Seoul" and "female". These keywords were then combined using Boolean operators to enable more specific searches using brand names, personnel and specialist industry terms identified in initial searches. A total of 1222 documents were reviewed.

A hermeneutic approach guided analysis of the documents. Documents relevant to the theme of gender-based tobacco industry activity in South Korea were indexed on a specially designed project database to enable the construction of an historical and thematic narrative. Documents were contextualised using secondary sources in the form of newspaper articles and academic journals. Interpretation of documents was corroborated by several authors, and the triangulation of findings was achieved through cross referencing of documents and the use of additional industry data sources. These supplementary data sources were obtained by systematic searches of the main industry publications, *Tobacco Journal International *and *Tobacco Reporter*, industry reports, database searches such as Medline, and on-line searching of Korean language websites using keywords related to female smoking and tobacco industry activities.

## Results

### Defining the female market in South Korea

Declining sales in established tobacco markets in North America and Europe since the 1970s have led TTCs to target new markets worldwide, including Asian countries, where higher rates of population growth, lower awareness of smoking and health issues, and weaker tobacco control regulations offer substantial prospects for growth. Although the initial focus of TTCs was to gain access to these countries, and win a share of the substantial market offered by male smokers, the potential for longer term growth offered by females was quickly recognised. In a 1979 report by Terry Hanby (Marketing Services, BAT), on expected future patterns of smoking prevalence by continent and gender, the potential for growth through increased female smoking rates was identified: " [A]lthough in many countries male incidence of smoking is plateauing or even declining, female incidence appears to be more robust often showing continued positive trends" [46]. Like the industry marketing campaigns beginning in the early twentieth century, marketing staff within BAT explicitly sought to link smoking with female emancipation as a symbol of equality and reliever of stress:

*Our main postulated explanations for it are the increasing liberation of females throughout the world and the greater quantities of disposable income which they are obtaining. The first of these factors increases the degree of stress placed upon women in their day to day lives and/or makes them more interested in adopting traditionally male habits such as smoking as symbols of their equality. The second factor, personal income, gives them the freedom to indulge in the habit. This move to sexual equality is liable to continue in the future and lead towards an equalisation of male and female incidence of smoking and consumption. Thus, although male consumption may plateau or even decline, female consumption will continue to rise *[46].

By the early 1990s, these ideas began to be applied to Asian women. As many countries in the region, including South Korea, experienced rapid economic growth, it was anticipated by industry analysts that the changing role of women would offer TTCs an opportunity to remain globally competitive:

Long-term international-tobacco outlook: Bullish....

We believe that a combination of factors has created a large one-time opportunity for global competitors who can move quickly:...

Easing trade restrictions in key Asian markets, notably Japan, Thailand, Korea, and Taiwan, should fuel export growth of 18% per year between 1992 and 1996....

*With rising social status and participation in the labor force, more Asian women have taken up smoking *[47].

In South Korea, the potential for TTCs to exploit rapid economic development and social change was immediately recognised. While Korean adult males had among the world's highest rates of smoking prevalence, there was a traditionally strong social stigma against female smoking. The country's significant economic growth from the 1960s, and integration into the global economy [48], brought with it widespread social change. As observed by the *International Herald Tribune*, smoking was once "taboo" for women in many Asian countries, but had become a sign of female emancipation [49,50]. In market research by PM Asia in 1990, it was observed that 14% of new smokers are female and that this market segment "should grow" [51]. At this point, females were seen as a critical component of the youth market which was regarded as "the prime development target market" [51] for foreign brands. BAT similarly recognised this potential, defining "starters" (new smokers) as young adults albeit skewed towards females [52].

TTCs began to focus attention on better understanding the female market in South Korea in the mid-1990s. The desire to increase the number of female smokers grew as smoking prevalence among adult Korean males began to decline. BAT predicted that, as sales volume declined among older adult males, this would be offset to some degree by increasing female smoking [53]. A 1995 Brown and Williamson (B&W)/BAT corporate plan stated that "Industry volume is expected to decline somewhat throughout the plan period due to the decline in smoking incidence of older male consumers. The rate of decline is partially offset by growth in female smoking incidence." In 1997, BAT research concluded that, while Korean society remained male-dominated, with men occupying an authoritarian role within the family, the traditional role of women was gradually changing [50]. This was due to women entering the workforce and learning to drive at increasing rates [54].

### Creating "broad permission to speak" [55]: Overcoming social and regulatory barriers to accessing the female market

The National Health Promotion Law Enforcement Ordinance, adopted in 1989, bans all tobacco advertising, marketing and sponsorship targeted at women and children including both print and broadcast media. Despite this restriction, smoking rates among Korean females has generally increased (Table 2), with evidence of the highest rises among females (17–19 years) [30,31]. Documents describe the strategies used by TTCs to circumvent this restriction. First, advertising of each cigarette brand, if not targeted at women or children, remained permitted in print media up to sixty times per year under the Tobacco Business Law Enforcement Ordinance (Article 9) [36]. Tobacco companies are also allowed to sponsor social, cultural, music and sporting events (other than events for women and children) using company names but not product names. A 1994 document describes how B&W adapted an international campaign for Capri/Finesse for use where targeting females is not permitted by using imagery of couples:[56,57]"Although obviously targeted to women, the campaign extension would also not be as overt in markets sensitive to female targeting" [57].

**Table 2:** Smoking prevalence of Korean females by age (1980–2003)

**Age**	**1980**	**1985**	**1990**	**1992**	**1994**	**1996**	**1999**	**2000**	**2001**	**2002**	**2003**
**20 – 29**	1.4	1.3	1.5	3.8	3.3	7.2	4.8	5.7	5.3	8.1	4.5

**30 – 39**	2.7	1.6	1.4	3.9	0.7	5.2	3.2	2.0	2.1	2.6	0.8

**40 – 49**	9.2	4.1	3.3	3.7	0.8	1.8	2.8	1.1	2.6	3.1	4.5

**50 – 59**	28.4	16.4	11.3	6.0	8.2	2.8	4.8	2.4	1.7	7.6	4.1

**60 +**	47.2	32.5	29.5	12.1	7.1	10.4	10.4	0	3.4	10.5	4.5

**Adjusted age**											

	12.6	8.0	7.7	5.1	3.5	5.3	4.4	3.0	3.1	6.0	3.5

Source: Korean National Health and Nutrition Survey, Seoul (2005)

Second, TTCs focused on retail distribution on venues which tended to be frequented by females. Documents describe the sale of female brands in restaurants, coffee shops, "event lunches" [53], bars, nightclubs and other popular gathering places for young girls and women. In a 1996 summary of the Korean market, BAT aimed to " [r]einforce positioning [of Finesse] as THE cigarette for independent Korean women" [58]. A key tactic for achieving this was "to expand its coffee shop program targeted at reaching the female audience" [58]. This expanded distribution through coffee shops was described as a means of exposing female smokers to *Finesse*, based on research showing that "the majority of purchasing and consumption of cigarettes by Korean females is made in coffee shops" [59].

Third, TTCs have used "trademark diversification" (TMD) to circumvent restrictions in order to promote selected brands to the female market. A 1999 BAT document defined TMD, sometimes known as brand stretching, as "the extension of a well-known trademark and its associated brand essence to a product or service unrelated to the one for which the trademark is traditionally associated" [60]. Examples of such practices include the use of tobacco branding on clothing, footwear, toiletries and holidays. The purpose of TMD, for TTCs, has been to circumvent growing regulatory restrictions:

*In a global environment of ever increasing restriction in the availability of traditional advertising media, parallel communications devices such as sponsorships and trademark diversification now represent the only major alternatives for tobacco marketers in a growing list of markets. Where traditional advertising media are available, their use should be maximized. However, in markets where these media are no longer available or are threatened in the foreseeable future, parallel communications should be seriously considered as part of a brand's marketing mix *[61].

In 1989, PM introduced the first consumer pack promotion in Korea for Virginia Slims which included a pocket size address book [62]. In 1990, BAT considered whether TMD would offer an improved opportunity over print media to communicate the stylish and feminine proposition of *Capri/Finesse*. A 1996 B&W report noted that, despite legal restrictions, KT&G had advertised its brand *Simple *in numerous magazines aimed at female readers. Strategies included the coupling of cigarettes with bottles of Chanel perfume [63], and the placement of advertisements in foreign language women's magazines available in South Korea.

Finally, TTCs used sports sponsorship to target certain age groups within the female market. In 1988, PM International noted that a tennis exhibition for Virginia Slims as a sponsorship would be acceptable, but cautions about associated perceptions noting "we have to be careful that we don't seem to be "targeting" females" [64]. At the same time, the industry was careful about obvious "targeting" of females. In 1991, BAT aimed to create a Kent Golf Sponsorship program targeted at higher-educated, male and females aged 25 years or older with above average incomes. Golf and *Kent *"were very image compatible...a creative natural fit for upscale sociability in a resort setting" [65].

### Developing products targeted at the female market

To capture the female market, TTCs undertook extensive research to develop products that would appeal to it. Findings identified female-specific styles and product preferences and specific types of packaging. For example, it was predicted that menthol flavoured cigarettes would increase slightly as a result of the growth in female smoking [53]. It was also observed that, "for younger adults starting to smoke and women, both...naturally find a lighter taste much more palatable and easier to enjoy" [66]. The marketing of "light" and "mild" cigarettes in South Korea, however, needed to take account of the unusual popularity of similar products among Korean males (Table 3). Market research found that light/mild and slim cigarettes were generally perceived by males in most Asian countries as too feminine [67]. In 1990, PM conducted the Korean Cigarette Market Study, a major market study among smokers (n = 1200) to gather information related to brand development in the Korean market. The study found that Korean smokers generally preferred "lighter" cigarettes. Recognising this, PM marketed Virginia Slims [68], originally developed for and targeted at female smokers in other countries, to Korean men using the tagline "For the Successful Man" [69]. At the same time, a 1995 BAT study observed that the popularity of "lights" was influenced by changing taste preferences and perceived health issues [66]. To appeal to females, TTCs saw "opportunities for super light and ultra light brands" [51].

**Table 3:** Percentage share of foreign brand market in South Korea (1994–1998)

	**Brand**	**1994**	**1995**	1996*	**1997**	**1998 (Jan)**
1	Mild Seven Light	39.0	45.3	26.6	24.9	31.8

2	Virginia Slims	19.6	18.8	26.0	32.9	20.2

3	Marlboro Light	11.0	9.0	12.8	10.3	7.5

4	Marlboro Medium	4.0	4.5	7.9	8.6	6.5

5	Dunhill Light	2.5	5.3	5.2	5.4	8.1

6	Marlboro Led	3.9	3.4	4.2	3.9	4.4

7	Virginia Super Slims	1.8	0.1	2.0	2.5	5.4

8	Finesse	3.9	3.1	3.4	2.5	3.9

9	Philip Morris Super Light	1.7	1.4	2.1	1.8	1.4

10	Salem Light	0.8	1.4	1.5	1.2	2.0

* The decline in the market share of several foreign brands between 1996 and 1998 can be explained by the Asian economic crisis during this period. The crisis led to a large outflow of investment and foreign currency from South Korea, increasing the price of foreign goods. Since 2000, the Korean economy has recovered and market share by foreign brands have steadily increased.

Source: Data from South Korea, Ministry of Finance and Economy, 1998 as quoted in Yoon YH. *Tobacco Market Opening and Tobacco Industry Analysis in South Korea*. Masters thesis, Korea University, Seoul; 1998.

Cigarette size was also identified as a characteristic that would appeal specifically to females. Based on a survey of Korean smokers aged between 18 and 54 years (450 male and 50 female), PM assessed the importance of different product themes and attributes by gender. Male participants rated thicker circumference cigarettes higher than female participants, and women rated cigarettes with less smoke and no smoke higher than men [70]. Brown & Williamson International (then BAT's American subsidiary) similarly aimed to develop brands for Korean females who favoured extra length and value-for-money [71].

Based on industry research, brands deemed to have feminine characteristics were developed and introduced in Korea from the late 1980s. BAT launched several brands, each aimed at a specific age group, led by *Finesse *(sold as *Capri *in other countries "with a female imagery campaign" [72]), "considered primarily (but not exclusively) for females" [73]. Marketed to "feminine, young, modern" [74] women,

*CAPRI/FINESSE is the U.S. International superslim cigarette offering the female smoker a statement of contemporary feminine style and a quality product. CAPRI is a fashion accessory. Target smokers are sophisticated, young adult females 21–35. The total CAPRI/FINESSE proposition projects a top quality image and makes the female smoker feel more like a woman *[73].

*Finesse *combined the growing worldwide popularity of "light" cigarettes with distinctive "superslim" dimensions:

*Capri/Finesse is targeted to the largest group of prospective superslim smokers, women. Prior to the introduction of superslims, brands positioned primarily to women held a small share of the world market...Capri/Finesse can be effective against those brands*.

*Finesse *smokers were described as younger (under 30 years of age), well-educated, more likely to be single, and having above average family incomes. As described by BAT, "Smoking Capri/Finesse makes a woman feel good about herself as a woman and allows her to make a style statement" [57]. Importantly, it was emphasised that this group represented a good share of starters and switchers [51]. South Korea, along with Italy and Japan, were projected as the largest markets for *Capri/Finesse *given high levels of support from consumers for a superslim product [75]. Volume gains for Finesse were expected to result from an increase in overall female smoking incidence [76].

By the late 1990s, BAT noted that improvements in taste and quality were needed to appeal more broadly to women. A 1997 BAT General Consumer Survey (GCS), a large, quantitative study (n = 500), investigated female-specific smoker usage, attitudes, behaviour, and brand images through face-to-face interviewing in places where females commonly smoked. The results indicated that, despite continued social stigma towards female smoking, female smokers were moving away from imported cigarette brands and showing signs of "maturity" [77]. Korean women wanted "to hear more about taste, quality, and a mild taste" and *Marlboro Lights *performed poorly on taste-related attributes [77].

In contrast with BAT, PMI targeted the female market with a single brand, a "light" version of *Virginia Slims*, the flagship female brand for the company worldwide. A 1989 PM report, *Korea Market Management*, recognized that the market for *Virginia Slims Light *(VSL) was growing at an impressive rate. To ensure continued growth, the company aimed to: (a) keep VSL in line with the trend towards lower tar and perceived product strength; and (b) introduce an ultra low version of the brand. At the same time, PM planned to launch a super slim product that would gain market share from *Finesse*, recognised as the only imported brand with a high share of female smokers (16%): "Since Finesse has a higher appeal to higher income female, a well-refined image for a Super slim brand with an acceptable product may have a chance to gain smokers from Finesse" [78]. By the mid 1990s, *Virginia Slims *and *Super Slims *together had become the second largest import brands behind Japan Tobacco's Mild Seven [79].

## Discussion

The historically high rates of smoking prevalence in South Korea, the world's highest at one time among adult males, spurred TTCs to seek market access in the 1980s. While gaining market share among male smokers was the initial aim, this analysis describes how TTCs have targeted girls and women as a promising source of future profits.

Tobacco control efforts by the Korean government and public health advocates to date have largely focused on reducing the number of male smokers. These efforts have been reported to have reduced male smoking rates, from 79% (1980) to 44% (2007) [80]. Smoking prevention programmes aimed at adolescents has also received increased attention since the mid 1990s [81]. However, increasing female smoking prevalence in South Korea, particularly among high school (17–19 years) girls, during the same period requires urgent and particular attention. As Choi Chang-mok of the Korean Anti-Smoking Institute argues, "the government is too obsessed in decreasing smoking rates of men. It's excessively political to only emphasize decreases in men's smoking rate since the recent trend is that smoking rate amid women and teenagers is on a significant increase" [82].

Following the Kobe Declaration, the WHO Framework Convention on Tobacco Control (FCTC) makes clear reference to the need for a gender perspective in strengthening tobacco control measures. As stated in Article 4, gender must be considered across all control policies adopted and implemented by signatories [83]. The findings of this paper raise a number of conclusions for strengthening tobacco control among Korean females.

First, the limited data available on female smoking prevalence and behaviour in South Korea must be urgently addressed. Data from the Korean National Health and Nutrition Survey (Table 2) suggests female smoking rates have fluctuated significantly between 1980 and 2003, with variations within age groups by year that are difficult to explain. There are also inconsistencies across different data sources which prevent clear understanding of smoking behaviour within specific cohorts by age, location, socio-economic group and other variables. There is a particular need to take account of substantial underreporting in a country where social stigma against female smoking remains strong. A study by Gallup Korea (part of the Gallup Organization which conducts public opinion polls) in 2007 finds 83.4% of Koreans believe that females should not smoke and that, perhaps unsurprisingly, 54.3% of Korean female smokers try to hide their behaviour. This suggests substantial underestimation of female smoking prevalence, believed to be around 17% [28]. As one industry analysis reported, "it is likely that the number of female smokers may be a lot bigger than the officially stated figure considering the majority of female smokers are still smoking in private. It is unusual to find a woman smoking on the street as it is taboo for women to smoke in public in South Korea" [13]. Accurate and comprehensive data across all age groups is a prerequisite to the development of an effective tobacco control strategy.

Second, fuller and more detailed data on female smoking behaviour would support more effective targeting of tobacco control measures. A KASH survey found that the smoking rate by high school girls (17–19 years) rose from 1.8% to 8.1% between 1988 and 1997, a finding supported by other surveys [32,84]. Smoking by adult females nearly doubled from 3.9% in 1989 to 6.7% in 1997 [85]. The 2005 Global Youth Tobacco Survey reports that 5.3% of middle and high school girls (8.1% among 13–15 years) currently smoke [86]. Newspaper reports suggest smoking rates among female middle school students increased from 0.9% in 2002 to 3.3% in 2006, and among female high school students from 2.4% in 1991 to 6.5% in 2005 (Korean Association of Smoking and Health: 2006, submitted) [87]. Fuller data across time on tobacco use by specific cohorts of Korean females should be used, alongside the findings of this paper, to identify and target vulnerable population groups with gender sensitive measures. Detailed analysis of female smoking behaviour should takes account of such factors as social context, patterns of consumption and brand preferences.

Third, documents reviewed in this paper support the need for stronger gender-based tobacco control measures that counter the strategic targeting of Korean females by TTCs. Product design associating smoking with body image and female emancipation, familiarly deployed elsewhere [2], have been extensively used in South Korea to appeal to female smokers. Industry sources confirm that BAT's *Finesse *was popular among young females during the first half of the 1990s [13], while *Esse Menthol *(KT&G) and *Virginia Superslims *(PM International) have dominated market share since the late 1990s, all designed to appeal to the female market. So-called "ultra light", "low tar" and "superslim" cigarettes have been particularly effective, suggesting certain brands offer a healthier or safer option, as well as appealing to female concerns about weight gain. Restrictions on the use of such descriptors, alongside public education on the fallacy of such claims, are needed. The creation and promulgation of certain aspirations and values to appeal to Korean females, in general, should be addressed by public health advocates through public disclosure and countermarketing.

Finally, the strategic targeting of females in South Korea by TTCs since market opening has occurred despite Article 14 of the National Health Promotion Act of 1989 which is intended to restrict advertising directed at women and children. These findings suggest that these restrictions as currently stipulated have been effectively circumvented and exploited by tobacco companies. In particular, the Act has not prevented the use of indirect marketing tactics, such as brand stretching, sponsorship of events, or the use of descriptors or product design that appeal to females. There is need for comprehensive tobacco control legislation in South Korea, commensurate with commitments under the Framework Convention on Tobacco Control, which bans all forms of tobacco advertising, marketing and promotion.

## Conclusion

Since the opening of the South Korean tobacco market in the late 1980s, females have been targeted by TTCs as an important source of future market growth and profitability. The rise in smoking rates among females within certain age groups since the late 1980s suggests that these efforts have been successful. The implementation of comprehensive tobacco control measures under the FCTC, from a gender perspective, is urgently needed to protect and promote the health of Korean women and girls.

## Abbreviations

BAT: British American Tobacco; B&W: Brown and Williamson; GATT: General Agreement on Tariffs and Trade; KT&G: Korean Tomorrow & Global; JTI: Japan Tobacco International; PMI: Philip Morris International; TTC: transnational tobacco company; USTR: United States Trade Representative.

## Competing interests

KL collaborated with the University of California, San Francisco and the Mayo Clinic in the Guildford Archiving Project which created the BAT Document Archive. KL has received funding for tobacco document research from the Rockefeller Foundation, Wellcome Trust, Cancer Research UK and Health Canada.

## Authors' contributions

KL, CCa and CCh undertook systematic searching and analysis of internal industry documents. KL and CCa drafted and revised the paper. SYL and CCh provided additional primary and secondary data, and commented on various drafts of the paper. GC and HK critically reviewed the manuscript.

## References

[B1] WHO (1999). Report of the fourth Meeting of the WHO Centre for Health Development. Kobe, Japan.

[B2] Ernster V, Kaufman N, Nichter M, Samet J, Yoon SY (2000). Women and tobacco: moving from policy to action. Bulletin of the World Health Organization.

[B3] Barbeau EM, Leavy-Sperounis A, Balbach ED (2004). Smoking, social class, and gender: what can public health learn from the tobacco industry about disparities in smoking?. Tob Control.

[B4] Amos A, Haglund M (2000). From social taboo to "torch of freedom": the marketing of cigarettes to women. Tob Control.

[B5] Kaufman N, Nichter M, Samet JYS (2001). The Marketing of Tobacco To Women: Global Perspectives. Women and the Tobacco Epidemic, Challenges for the 21st Century.

[B6] Greaves L, Jategaonkar N, Sanches S (2006). Turning a New Leaf: Women, Tobacco, and the Future.

[B7] Sarna L, Bialous SA (2004). Why tobacco is a women's health issue. Nurs Clin North Am.

[B8] WHO (1999). "'Kobe Declaration' Calls for a half to the tobacco menace among women and children". WHO/71.

[B9] WHO (1999). 'Young Women's Smoking Crisis' set to hit Asia. Press Release.

[B10] Bansal R, John S, Ling PM (2005). Cigarette advertising in Mumbai, India: targeting different socioeconomic groups, women, and youth. Tob Control.

[B11] Morrow M, Barraclough S (2003). Tobacco control and gender in Southeast Asia. Part I: Malaysia and the Philippines. Health Promot Int.

[B12] Ho M, Shi Y, Ma S, Novotny TE (2007). Perceptions of tobacco advertising and marketing that might lead to smoking initiation among Chinese high school girls. Tob Control.

[B13] Euromonitor (2005). Tobacco in South Korea.

[B14] US GAO (1992). Advertising and Promoting US Cigarettes in Selected Asian Countries. Washington DC.

[B15] Lambat I (2006). Paradigm Shift. Tobacco Reporter.

[B16] Barton HC (1993). New Business Development Strategy. British American Tobacco. Bates No. 2027000015-202700016. British American Tobacco.

[B17] Taylor A, Chaloupka FJ, Guindon E, Corbett M, Jha P, Chaloupka FJ (2000). The impact of trade liberalization on tobacco consumption. Tobacco Control in Developing Countries.

[B18] US GAO (1990). Trade and Health Issues: Dichotomy Between U.S. Tobacco Export Policy and Antismoking Initiatives. Report to Congressional Requesters. Washington DC.

[B19] Yoon YH (1998). Tobacco Market Opening and Tobacco Industry Analysis in South Korea.

[B20] Yoo SJ (2006). BAT Korea aims to surpass 16% market share. Korea Herald.

[B21] Lee K, Kinh HV, MacKenzie R, Gilmore AB, Minh NT, Collin J (2008). Gaining access to Vietnam's cigarette market: British American Tobacco's strategy to enter "a huge market which will become enormous". Global Public Health.

[B22] Lee K, Gilmore AB, Collin J (2004). Breaking and re-entering: British American Tobacco in China 1979–2000. Tob Control.

[B23] Lee K, Collin J (2006). "Key to the future": British American tobacco and cigarette smuggling in China. PLoS Med.

[B24] Knight J, Chapman S (2004). "Asian yuppiesare always looking for something new and different": creating a tobacco culture among young Asians. Tob Control.

[B25] Assunta M, Chapman S (2004). "The world's most hostile environment": how the tobacco industry circumvented Singapore's advertising ban. Tob Control.

[B26] Knight J, Chapman S (2004). "Asia is now the priority target for the world anti-tobacco movement": attempts by the tobacco industry to undermine the Asian anti-smoking movement. Tob Control.

[B27] MacKenzie R, Collin J, Sriwongcharoen K, Muggli ME (2004). "If we can just 'stall' new unfriendly legislations, the scoreboard is already in our favour": transnational tobacco companies and ingredients disclosure in Thailand. Tob Control.

[B28] (2007). Gallop Korea. Investigating the Actual Condition of Smoking in South Korea.

[B29] Juon HS, Shin Y, Nam JJ (1990). Cigarette smoking among Korean adolescents: prevalence and correlates. Adolescence.

[B30] Weissman R, Hammond R (1998). International Tobacco Sales. Foreign Policy in Focus.

[B31] Shafey O, Dolwick S, Guindon GE (2003). Tobacco Control Country Profiles.

[B32] Han S, Choe MK, Lee MS, Lee SH (2001). Risk-taking behaviour among high school students in South Korea. Journal of Adolescence.

[B33] Jee SH, Lee J, Kim IS (2006). Smoking attributable mortality among Korean adults: 1970–2020. Korean Journal of Epidemiology.

[B34] Ha BM, Yoon SJ, Lee HY, Ahn HS, Kim CY, Shin YS (2003). Measuring the burden of premature death due to smoking in Korea from 1990 to 1999. Public Health.

[B35] Kang HY, Kim HJ, Park TK, Jee SH, Nam CM, Park HW (2003). Economic burden of smoking in Korea. Tob Control.

[B36] (1995). Korean Ministry of Health: National Health Promotion Act, Article 14, Seoul.

[B37] dongA.com (2007). Domestic Tobacco Brands Losing Market Share. 25 July 2007. The Dong-A Ilbo.

[B38] MacKenzie R, Collin J, Lee K (2003). The Tobacco Industry Documents: An Introductory Handbook and Resource Guide for Researchers.

[B39] Malone RE, Balbach ED (2000). Tobacco industry documents: treasure trove or quagmire?. Tob Control.

[B40] Bero L (2003). Implications of the tobacco industry documents for public health and policy. Annu Rev Public Health.

[B41] Balbach ED, Gasior RJ, Barbeau EM (2002). Tobacco industry documents: comparing the Minnesota Depository and internet access. Tob Control.

[B42] LeGresley EM, Muggli ME, Hurt RD (2005). Playing hide-and-seek with the tobacco industry. Nicotine Tob Res.

[B43] Collin J, Lee K, Gilmore AB (2004). Unlocking the corporate documents of British American Tobacco: an invaluable global resource needs radically improved access. Lancet.

[B44] Muggli ME, LeGresley EM, Hurt RD (2004). Big tobacco is watching: British American Tobacco's surveillance and information concealment at the Guildford depository. Lancet.

[B45] Lee K, Gilmore AB, Collin J (2004). Looking inside the tobacco industry: revealing insights from the Guildford Depository. Addiction.

[B46] Hanby TC (1979). Basic Data Free World. 22 March 1979. British American Tobacco. Bates No. 104751554-1559.

[B47] Black GD (1992). Bernstein Research Weekly Notes – Special Tobacco – Industry Issue. 9 December 1992. British American Tobacco. Bates No. 500098715-8751.

[B48] Harvie C, Lee HH (2003). Export-led industrialisation and growth: Korea's economic miracle, 1962–1989. Australian Economic History Review.

[B49] Shenon P Cigarette Markers See Future It's in Asia. 17 May 1994. British American Tobacco. Bates No. 202058367.

[B50] BAT (1997). Research International Study Comissioned by CORA. 20 March 1997. British American Tobacco. Bates No. 321208326-573.

[B51] PM (1990). Korean Cigarette Market Study. Phillip Morris. Bates No. 2504034023-2504034055 (2504034026).

[B52] PM (1993). General Consumer Tracking Survey Korea. Phillip Morris Asia. Bates No. 2504026687-2504026772.

[B53] BAT Brown & Williamson Tobacco Corporation 1996–1998 Plan. Bates No. 14323-388 (14355).

[B54] BAT The Effect of Tobacco Advertising, Sponsorship and Trademark Diversification Restrictions on the Consumption of Tobacco Products A Summary of the Literature. British American Tobacco. Bates No. 321454092-287.

[B55] Morgan B (1997). British-American Tobacco Opinion Benchmark Study, Management Summary. Bates No. 700772115-2157 (700772133).

[B56] BAT (1991). 1991 1st Quarter Update Capri/Finess. British American Tobacco. Bates No. 500188301-8302.

[B57] SDZ (1990). Capri/Finesse – Brand Review. British American Tobacco. Bates No. 201813077-3093.

[B58] BAT (1996). BAT America-Pacific Region – Regional Plan 1997–1999. British American Tobacco. Bates No. 800123509-3658 (800123510).

[B59] Bexon RL (1996). [Letter to Ulrich Herter]. British American Tobacco. Bates No. 800262266-2275.

[B60] BAT (1999). World Investment Company, An Explanation of the Trademark Diversification Activities of British-American Tobacco. Bates No. 322048676.

[B61] BAT (1991). Confidential, Trademark Diversification. British American Tobacco. Bates No. 502594945-4952.

[B62] PM (1990). Virginia Slims Below-the-Line Programs Update Hong Kong Taiwan Japan Korea. Phillip Morris. Bates No. 2500152276-2329.

[B63] Bexon RL (1996). [Letter to Ulrich Herter]. British American Tobacco. Bates No. 800104735-4745.

[B64] PM (1988). Special Events. Phillip Morris. Bates No. 2504048307-8308.

[B65] BAT (1999). Kent Sponsorship. British American Tobacco. Bates No. 202227483-7485.

[B66] BAT (1995). The Lights Phenomenon. 18 December 1995. British American Tobacco. Bates No. 800178765.

[B67] Market Behaviour (Hong Kong) L Project Galactic, A Report. British American Tobacco. Bates No. 303502423-2528 (303502488).

[B68] Toll BA, Ling PM (2005). The Virginia Slims identity crisis: an inside look at tobacco industry marketing to women. Tob Control.

[B69] Dewhirst T, Lee W, Fong G, Ling P (2007). International advertising and the gender of nations: A case study of Virginia Slims advertising in the United States, Japan and Korea. 8th Asia Pacific Conference on Tobacco or Health; Taipei, Taiwan.

[B70] PM (1990). Korean Concept Study. Philip Morris. Bates No. 2057094948-4991.

[B71] BAT Management Overview. Financial report. British American Tobacco. Bates No. 202754576-656.

[B72] SDZ (1990). Capri/Finesse – Brand Review. British American Tobacco. 25 January 1990. Bates No. 201813077-3093 (201813079).

[B73] Tonge J (1991). Korea: Finesse Ad Research (Int'l 1991-5) – Final Report. Brown & Williamson. Bates No. 464527929-464527947.

[B74] (1998). 1997 General Consumer Survey: Korea Female Sample. Brown & Williamson. Bates No. 210243698-3942. Asia Market Intelligence.

[B75] BAT (1992). US International Brands and Strategic Review. British American Tobacco. Bates No. 201817078-7101.

[B76] BAT Korea Market Overview. British American Tobacco. Bates No. 800058927-933.

[B77] Ha KP, Thompson K, Harrison R (1998). 1997 General Consumer Survey: Korea – Female Sample. Brown & Williamson. Bates No. 210243698-3942.

[B78] PM (1989). Project Nova 1& 2. Phillip Morris. Bates No. 2504051101-1113.

[B79] BAT (1995). Quarterly Competitive Activity Report Quarter Three 1995. British American Tobacco. Bates No. 500030963-0980.

[B80] (2007). Male Smoking Rate Drops to Under 50%. Chosun Ibo.

[B81] Park E (2006). School-based smoking prevention programs for adolescents in South Korea: a systematic review. Health Education Research Theory & Practice.

[B82] Park CA (2007). Anti-Smoking Achievements Exaggerated. Korea Times.

[B83] WHO/IDRC (2007). Gender and tobacco control: a policy brief.

[B84] KASH (2004). A National Survey on Smoking among Middle- and High School Students.

[B85] WHO (2002). Smoking Statistics. Fact Sheet.

[B86] Warren CW, Jones NR, Peruga A, Chauvin J, Baptiste J-P, Costa de Silva V, el Awa F, Tsouros A, Rahman K, Fishburn B (2008). Global Youth Tobacco Surveillance, 2000–2007. Surveillance Summaries.

[B87] Smoking Gaining Popularity Among Korean Teens. Chosun Ibo.

